# Role of enamel and dentin on color changes after internal bleaching associated or not with external bleaching

**DOI:** 10.1590/1678-7757-2020-0511

**Published:** 2020-12-16

**Authors:** Tauan Rosa SANTANA, Rafaella Mariana Fontes de BRAGANÇA, Ana Claudia Conceição CORREIA, Isadora de Melo OLIVEIRA, André Luis FARIA-E-SILVA

**Affiliations:** 1 Programa de Pós-graduação em Odontologia Universidade Federal de Sergipe AracajuSE Brasil Programa de Pós-graduação em Odontologia, Universidade Federal de Sergipe, Aracaju, SE, Brasil.; 2 Departamento de Odontologia Universidade Federal de Sergipe AracajuSE Brasil Departamento de Odontologia, Universidade Federal de Sergipe, Aracaju, SE, Brasil.

**Keywords:** Dental enamel, Esthetics, dental, Dentin, Hydrogen peroxide, Tooth bleaching

## Abstract

**Objective:**

To evaluate the effect of the association between external and internal tooth bleaching on color changes in dentin and enamel, individually or recombined, previously stained with triple antibiotic paste (TAP).

**Methodology:**

Forty enamel-dentin specimens from bovine incisors were separated into ten blocks according to similarity in their whiteness index (WID). Three specimens within each block were stained by dentin exposure to TAP, and the remaining specimen was used as control to estimate color changes. Specimens were sectioned to separate tissues, and dentin and enamel colors were measured individually and after being recombined. Alterations in color (CIEDE2000 - ΔE_00_) and translucency parameter (TP) resulting from staining were estimated by color difference between stained and control specimens. The contribution of each tissue to the color change (CTCC) was also calculated. Non-sectioned stained specimens were bleached by applying sodium perborate on dentin, associated or not with 35% hydrogen peroxide on enamel. Color changes caused by bleaching procedures were estimated and data were analyzed using the paired t-test or Two-way repeated measures ANOVA.

**Results:**

TAP caused more pronounced changes in dentin, but enamel color was also affected. Both protocols presented a similar ΔE_00_, and dentin showed the greater color change. After exposure to TAP, we observed a reduction in WID; WID values were the same for bleached and control specimens regardless of protocol. We found no significant effect of substrate and bleaching technique on TP. Enamel played a more critical role in color changes caused by either staining or bleaching procedures.

**Conclusion:**

Enamel color played a greater role on tooth color changes than dentin. External and internal bleaching association did not improve bleaching effect on specimens stained with TAP.

## Introduction

Tooth color results from the combination between dentin and enamel individual color and optics characteristics.^1^ Being more chromatic, dentin is the main dental tissue affecting overall tooth color;^2 , 3^ however, enamel surface characteristics affect light transmission, modifying tooth color.^3^ Enamel is a chromatic translucent tissue that modifies the underlying dentin appearance,^1 , 4 , 5^ so changes within the color or translucency of both of these dental hard tissues may cause tooth discoloration.

Despite the high success of root canal therapy, endodontic interventions may cause tooth discoloration not only due to dental trauma and pulp necrosis, but also due to some filling materials, sealers, and intracanal medications.^6 - 8^ Triple antibiotic paste (TAP) was used for a long time as an intracanal medication. However, this medication was reported to entail significant tooth discoloration^9 , 10^ attributed to the presence of minocycline,^11 , 12^ which lead researchers to recommend its replacement by amoxicillin to avoid discoloration.^13^

Internal bleaching is a relatively safe and conservative treatment for discoloring endodontically treated teeth. It consists of applying the bleaching agent closer to dentin, as this tissue is strongly related to color changes. Each tissue (dentin and enamel) contribution to overall tooth color changes in bleaching procedures remains controversial.^1 , 3 , 14 , 15 , 16^ Studies reported that enamel opacity increase due to the action of hydrogen peroxide is the major responsible for bleaching effect,^1 , 17 , 18^ indicating that associating both internal and external bleaching procedures could optimize bleaching effect.^19^

Considering that, this study aimed to evaluate the effect of the association between external and internal tooth bleaching on color changes in dentin and enamel, individually or recombined, previously stained with TAP. Our null hypotheses were that (1) associating external and internal bleaching techniques would not affect bleaching effectiveness and (2) Enamel and dentin contribution to overall tooth color change are not different.

## Methodology

### Specimen preparation

Forty enamel-dentin specimens (6x6x2 mm thickness) were sectioned from bovine incisors using a water-cooled low-speed saw and a double-sided diamond disk (#7020; KG Sorensen, Barueri, SP, Brazil). Sample size was determined according to the dependent variable “contribution of each tissue to the color change” (CTCC). Acceptability threshold of ΔE_00_ = 2.66^20^ was used as the minimum detectable difference in means and calculated based on the mean and standard deviation (1.66) reported by a previous study assessing CTCC after bleaching procedures,^1^ adopting a 80% statistical power and 5% type I error. Buccal enamel and opposite dentin were abraded with SiC sandpaper (#320) until obtaining a 1-mm thickness for each tissue. Enamel surface was polished using #600 and #1200 SiC sandpapers. Specimen dimensions were verified using digital calipers.

### Baseline measurements

Specimens initial color was measured with a portable spectrophotometer (Easyshade Compact V5, Vita-Zahnfabrik, Bad Säckinge, Germany) positioned on enamel surface. As the spectrophotometer tip diameter (6 mm) was similar to specimens surface dimensions, no index was used. Color was measured in triplicate, with specimens placed over a white background (L* = 94.5; a* = -0.9; and b* = 2.9). The parameters L* (lightness), a* (red-green axis), and b* (yellow-blue axis), defined by the International Commission on Illumination ( *Commission Internationale de L’Eclairage* –CIE), were recorded and used to calculate whiteness index (WID) according to the following formula:^21^

WID=0.551×L−2.324×a−1.1×b

Specimens which WID values ranged more than the standard deviation from specimen averages were replaced. Then, they were separated into ten blocks (n=4) according to similarity in their WID values.

### Staining procedure

Three specimens of each block were randomly allocated to undergo staining procedures; the remaining were used as control to estimate color changes caused by the procedure. The dentin surface opposite to buccal surface was exposed to TAP (ciprofloxacin, metronidazole, and minocycline) and the other faces were protected with wax. Before staining procedure, the exposed dentin was acid-etched with 35% phosphoric acid for 15 s, rinsed, and air-dried. Then, TAP was mixed, applied onto the exposed dentin, and left undisturbed for 15 days. After this period, wax was removed and specimens were washed in running water and kept in distilled water at 37°C during one week for rehydration. Control specimens were kept in distilled water throughout staining period.

One stained specimen and the control specimen were sectioned to separate enamel from dentin using a diamond saw (Extec; Enfield, CT, USA) in a cutting machine (Isomet low-speed, Buehler; Lake Bluff, IL, USA). The color of each tissue was individually measured as above described, and WID values were calculated by placing the spectrophotometer tip on either enamel or dentin buccal surface (not exposed to TAP). Tissues were also recombined by applying an optical glycerin solution (refractive index ≈ 1.47) between them, and recombined specimens color was measured by placing the spectrophotometer tip on enamel surface. Color changes caused by staining procedure were estimated based on the color difference between stained and control specimens using the CIEDE2000 equation:^22^

$$\Delta E_{00}=\sqrt{{\left(\frac{\Delta L^{\prime }}{K_{L}S_{L}}\right)}^{2}+{\left(\frac{\Delta C^{\prime }}{K_{C}S_{C}}\right)}^{2}+{\left(\frac{\Delta H^{\prime }}{K_{H}S_{H}}\right)}^{2}+R_{T}\frac{\Delta C^{\prime }}{K_{C}S_{C}}\frac{\Delta H^{\prime }}{K_{H}S_{H}}}$$

where ΔL’ represents changes on lightness, ΔC’ on chroma, and ΔH’ on hue; S_L_, S_C_, and S_H_ are the weighting functions for each component, and R_T_ the interactive term between chroma and hue differences.

Specimens color was also measured on a black background (L* = 21.6; a* = -0.1; and b* = -0.4) and the translucency parameter (TP) of enamel, dentin, and recombined specimens were calculated using the following formula:

$$TP=\sqrt{{\left(L_{\text{white}}-L_{\text{black}}\right)}^{2}+{\left(a_{\text{vhite}}-a_{\text{black}}\right)}^{2}+{\left(b_{\text{white}}-b_{\text{blach}}\right)}^{2}}$$

Changes on TP (ΔTP) caused by the staining procedures were estimated by subtracting TP values for stained specimens from those of control specimens.

**Figure 1:**
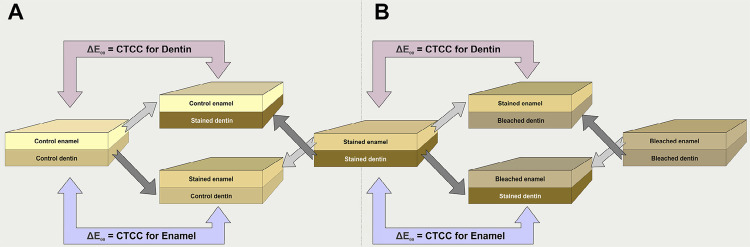
Schematic illustration of tissue recombination for measuring the contribution of tissues to color change (CTCC). A and B are the recombinations used to measure the effects of staining and bleaching procedures, respectively. Color data measured by the spectrophotometer were used to shade figures. Blocks upper color is that measured from combined specimens

Enamel and dentin specimens were recombined to measure CTCC in each tissue. Enamel CTCC was estimated based on color differences (ΔE_00_) between recombined stained enamel and control dentin and recombined control tissues. Dentin CTCC was estimated based on the recombination of control enamel and stained dentin ( Figure 1A ).

### Bleaching procedure

Non-sectioned specimens underwent dental bleaching procedures. Both specimens were bleached by applying 20% sodium perborate (Whiteness Perborato, FGM, Joinvile, SC, Brazil) on dentin, simulating internal bleaching. After five days, perborate was replaced and left undisturbed for the same period. One specimen (associated bleaching) per block underwent external bleaching, using a 35% hydrogen peroxide-based bleach (Whiteness HP Maxx, FGM, Joinvile, SC, Brazil), immediately before each perborate application, resulting in two 45-min applications. After the bleaching procedures, all specimens were kept in distilled water for one week.

Bleached specimens were sectioned to separate enamel and dentin, as above described. The color of each tissue was measured individually and combined, and ΔE_00_ was calculated based on stained specimens color difference. Changes in TP caused by the bleaching procedures were also calculated by subtracting bleached specimens TP (both techniques) from stained specimens TP. CTCC for the bleaching effect was calculated by combining bleached and stained specimens ( Figure 1B ).

### Statistical analysis

Data presented normal distribution (Shapiro-Wilk test, p>.05) and equality of variance (Levene’s tests, p>0.05). Data on ΔE_00_ and ΔTP measured after staining procedures were analyzed by the one-way repeated measures ANOVA based on the factor ‘substrate’. After bleaching procedures, data were analyzed by two-way repeated measures ANOVA based on ‘bleaching technique’ and ‘substrate’ (repetition factor). Data on WID values were analyzed by the two-way repeated measures ANOVA based on ‘treatment’ (stained specimens, bleached by internal or associated technique, and control) and ‘substrate’ (repetition factor). Data on CTCC measured after staining procedures were analyzed by the paired t-test, while data measured after bleaching procedures were analyzed by the two-way repeated measures ANOVA to assess ‘bleaching technique’ and ‘substrate’ (repetition factor). A significance level of 95% was set for all analyses, which were performed using the SigmaPlot 12.0 statistical software package (Systat Software Inc., Chicago, IL, USA).

## Results

Table 1 presents the overall color differences (ΔE00) according to substrate and bleaching technique. After staining procedure, one-way repeated measures ANOVA showed a significant difference among substrates (p<.001). Dentin showed the more pronounced color change, without significant difference between enamel and combined tissues. After bleaching procedure, two-way repeated measures ANOVA showed difference between substrates (p=.004) regardless of the technique used (factor ‘bleaching technique’, p=.729; combination, p=.731), and dentin showed the more pronounced color change, without differences between enamel and combined substrates.

**Table 1:** Means (standard deviations) of ΔE00 according to substrate and bleaching technique (n = 10)

Substrate	Staining (1)	Bleaching (2)	
		Associated	Internal
Dentin	25.2 (9.2)^A^	17.2 (5.7)^Aa^	19.8 (7.5)^Aa^
Enamel	9.5 (7.1)^B^	12.4 (5.0)^Ba^	12.2 (6.3)^Ba^
Combined	8.6 (6.0)^B^	11.6 (8.0)^Ba^	11.0 (7.3)^Ba^

Data analyzed by one-way (staining) or two-way (bleaching) repeated measured ANOVA. Distinct letters (uppercase for lines, lowercase for columns) indicate significant differences at Tukey`s test (p < .05). 1. Color change from control. 2. Color change from stained specimens.

Table 2 shows whiteness index (WID) results. Two-way repeated measures ANOVA showed that both bleaching technique (p<.001) and substrate (p<.001) affected WID values, and their combination was also significant (p<.001). Except for internally bleached specimens (no difference), dentin presented the lowest WID values and enamel and combined specimens presented a similar color. Staining reduced WID values for all substrates, but reduction was more pronounced in dentin. Both bleaching techniques reached WID values similar to those observed in control regardless of the substrate. In dentin, both techniques reached higher WID values than those measured in stained specimens. Bleached and stained enamels were only different regarding associated technique. By evaluating combined specimens, we found no statistical difference between both bleaching techniques and stained specimens.

**Table 2:** Means (standard deviations) of WID according to substrate and bleaching technique (n = 10)

Substrate	Control	Stained	Bleaching	
			Associated	Internal
Dentin	3.1 (6.6)^Ba^	-31.7 (12.0)^Bb^	3.6 (5.1)^Ba^	6.1 (10.7)^Aa^
Enamel	16.4 (2.9)^Aa^	0.4 (15.1)^Ab^	13.1 (9.2)^Aa^	7.4 (8.7)^Aab^
Combined	18.4 (3.2)^Aa^	4.6 (11.2)^Ab^	13.8 (8.4)^Aab^	9.1 (6.9)^Aab^

Data analyzed by two-way repeated measured ANOVA.Distinct letters (uppercase for lines, lowercase for columns) indicate significant differences at Tukey`s test (p < .05). WID – whiteness index.

Table 3 shows the contribution of each tissue to the color change (CTCC) results. Regarding staining procedure, enamel played a more crucial role in tooth color change than dentin (p=.040). As for bleaching procedures, two-way repeated measures ANOVA showed that only the factor ‘substrate’ (p=.015) affected CTCC; neither ‘bleaching technique’ (p=.633) nor combination (p=.659) were significant. Regardless of the bleaching technique used, enamel played a more crucial role in color changes than dentin.

**Table 3:** Means (standard deviations) of CTCC according to substrate and bleaching technique (n = 10)

Substrate	Staining (1)	Bleaching (2)		
		Associated	Internal	Pooled averages*
Dentin	5.2 (3.2)^B^	7.4 (4.7)	7.9 (3.4)	7.7 (4.0)^B^
Enamel	10.4 (5.1)^A^	9.9 (5.3)	11.2 (5.5)	10.6 (5.3)^A^

Data analyzed by paired T-test (Staining) or two-way repeated measured ANOVA (Bleaching). Distinct letters indicate significant differences at Tukey`s test (p < .05). CTCC: contribution of tissue to color change. 1. Calculated by ΔE00 from control. 2. Calculated by ΔE00 from stained specimens. * Average of means from different levels of the independent variable.

**Figure 2:**
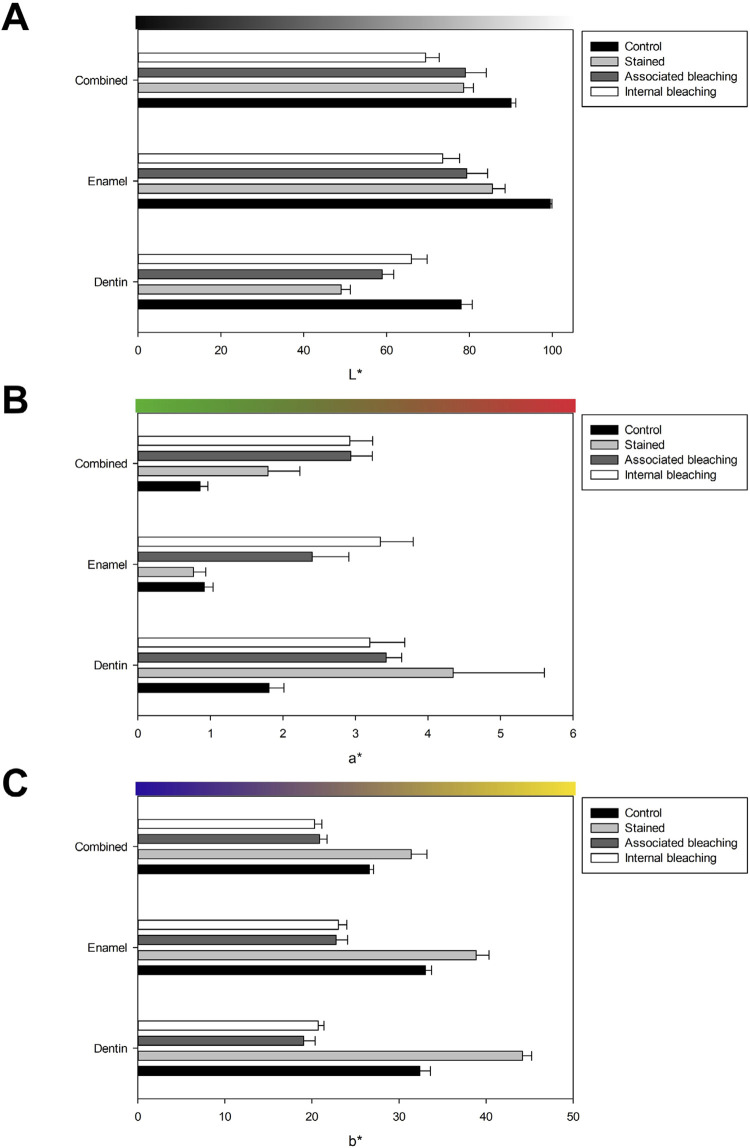
Color parameters behavior due to staining and bleaching procedures measured on dentin, enamel, or combined tissues. (A) – L* parameter measuring lightness, (B) – a* parameter for red-to-green axis, and (C) – b* parameter for yellow-to-blue axis

Figure 2 describes the behavior of L*, a*, and b* parameters throughout the study. After the staining procedure, dentin and enamel lightness decreased, both individually and combined ( Figure 2A ). Both bleaching techniques enhanced L* values in dentin but did not reach values observed in control. Enamel and combined substrates presented lower or equal post-bleaching values. Control specimens had the lowest a* values of all substrates (such as stained specimens in enamel), as shown in Figure 2B . Staining reduced redness in all substrates compared to control specimens, except in enamel. Both bleaching techniques reduced redness in dentin but increased a* values in enamel and combined substrates. All substrates behaved similarly regarding b* ( Figure 2C ), which were more yellow after staining procedures and became bluer after both bleaching techniques.

**Figure 3:**
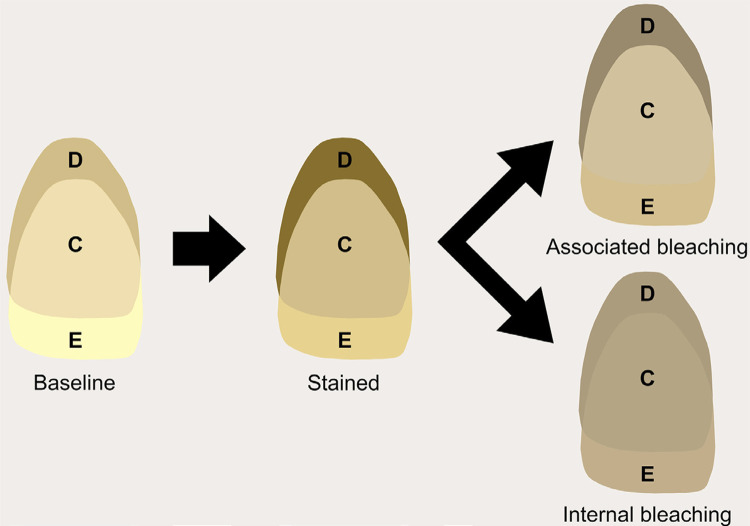
Illustrative image of color changes after staining and bleaching procedures. Substrates and background colors were painted using L*, a*, and b* parameters. D – dentin, C, combined, and E – enamel

**Figure 4:**
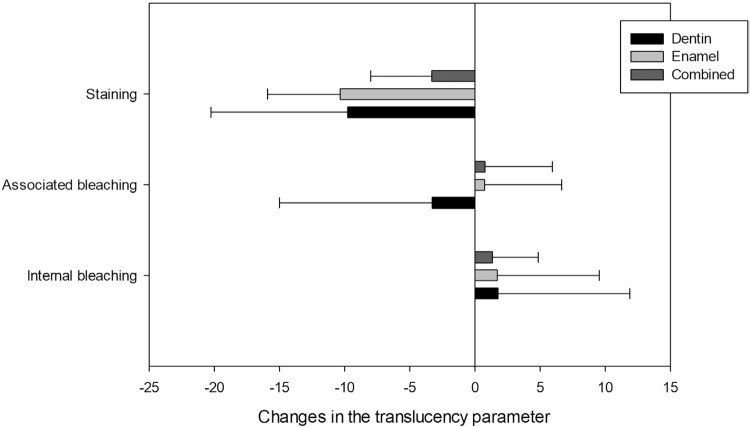
Changes on translucency parameters of dentin, enamel, and combined tissues caused by staining or bleaching procedures

We developed an experimental design scheme and created images with L*, a*, and b* values for each substrate using CorelDraw Graphics Suit X8 (Corel Corporation, Ottawa, ON, Canada) to visualize color changes ( Figure 3 ). Figure 4 shows ΔTP results. After the staining procedure, one-way repeated measure ANOVA found no significant difference among substrates (p=.140). Two-way repeated measures ANOVA also verified that neither ‘substrate’ (p<.633) nor ‘bleaching technique’ (p<.363) affected ΔTP values (interaction between the factor, p=.٥٦٢).

## Discussion

Both bleaching techniques evaluated in this study reached similar ΔE_00_ values for enamel, dentin, or combined tissues and changed WID values only in combined tissues, confirming our first null hypothesis. We found combined specimens to present close ΔE_00_ and WID values to those measured in enamel, suggesting that color change within enamel had a greater effect on combined tissues ultimate color. This is confirmed by the fact that enamel showed higher CTCC values than dentin, rejecting our second null hypothesis.

As most studies in this subject assess bleaching effect on teeth without approaching individual effects on enamel and dentin,^24 - 27^ the exact mechanism explaining peroxides action on each tooth substrate bleaching has not yet been fully elucidated.^5 , 18 , 23^ Although both substrates present similar compositions, differences within their inorganic and organic components ratio modify their interaction with light, such as reflection, transmission, refraction, and scattering.^4^ We separated specimens hard dental tissues after staining and bleaching procedures to assess color changes within each substrate both separately and in recombination. A prior study has employed this method^1^ and calculated color changes by comparing different specimens. This procedure requires that only specimens with similar colors at baseline are used to calculate color change. In that study, CTCC was calculated by comparing color differences between specimens from a same tooth (premolar).^1^ In our study, specimens were divided into ten blocks (corresponding to n) with four specimens each (corresponding to the number of treatments), all with similar WID values at baseline. Color changes were calculated based on color differences between specimens within the same group. Although this method does not enable an exact color change measurement, it does allow us to estimate these changes and compare each tissue effect on the overall tooth color.

Tooth bleaching is a dynamic process not fully elucidated. Peroxides and their subproducts react with both dentin and enamel to yield whiter teeth. Recently, peroxides bleaching effect on dentin was proved to be associated with peroxide interaction with phosphoproteins or, more specifically, with benzene ring oxidation on aromatic amino acid complexes.^28 , 29^ Some studies report that enamel increased opacity due to its organic content oxidation contributes to tooth bleaching.^23^ However, both internal and external bleaching techniques caused no clinically significant changes in enamel translucency parameter (TP) values, as their averages were lower than the acceptability threshold (TP=4.33).^30^ We also verified no significant ΔTP values for dentin and recombined tissues. In fact, data on TP presented high variability and seemed unreliable, which may be explained by the dental spectrophotometer used. The Easyshade was developed to read teeth color, which contains yellow and yellow-red colorations.^31^ Although this device has been used to calculate the TP of dental materials,^32 - 34^ placing a thin specimen (≈ 1.0 mm) over a black background results in a grayish color, compromising Easyshade’s ability to accurately measure specimens color.

After staining procedure, dentin presented the highest ∆E_00_ values and the greatest changes within WID. This may be explained by the fact that triple antibiotic paste (TAP) was only applied on dentin inner surface while specimens surrounding walls and enamel surface were protected with wax, simulating discoloration caused by an endodontic treatment using TAP. Minocycline (a component of TAP) is an antibiotic derived from tetracycline that binds to calcium and form an insoluble complex, resulting in a darker substrate.^11^ Before applying TAP, dentin was etched to remove the coarse smear layer from flattening procedures performed with SiC sandpapers. We expected no significant reduction in dentin calcium content, as the 35% phosphoric acid application for 15 s over dentin only demineralizes approximately 2.0 µm of this tissue.^35^ Considering that, we expected more pigment within the dentin matrix and an increased bleaching effect, as a darker substrate tends to achieve better bleaching outcomes. Interestingly, enamel played a greater role on overall color changes than dentin, besides reaching the lowest color change values. This may be justified by the spectrophotometer tip, which was placed on enamel during combined specimens color measurement. Unlike dentin, no overlay substrate modified enamel color, and even a slight change within this tissue modified the combined specimen color, which is supported by the fact that enamel color changes values are closer than dentin values to those found in combined specimens.

Although tooth dentin is thicker than enamel, this study used both tissues at the same thickness (1 mm), resulting in specimens with an overall thickness of 2 mm, which may help explain the lack of color change differences between the two bleaching techniques. Thin tissues might enable peroxide and its subproducts to penetrate throughout the entire specimen, causing peroxide application on enamel to have no significant effect on reactive oxygen specimens availability in either tissue. The 1-mm thickness of bovine incisors dentin was a limitation of this study; using thicker dentin would hinder specimen thickness standardization due to the high variability in pulpal chamber volume of bovine incisors.

Besides overall color change, analyzing modifications within each parameter is crucial to understand staining and bleaching effects on color. As expected, the staining procedure reached lower lightness values than those measured in control specimens. Regardless of the protocol, bleaching procedure increased lightness in dentin, but reached moderate L* values in enamel. We found no significant ΔTP; yet, the highest L* after bleaching procedures may be related to the white background reduced visualization, indicating an increase in enamel opacity. After staining, we verified that specimens were more red and yellow, especially dentin. These findings confirm TAP ability to increase dentin chromaticity^27^ and minocycline ability to reach enamel, modifying its color (increased yellowness). Dental bleaching reduced yellowness in both dentin and enamel, as expected. In turn, peroxide reduced dentin redness but increased enamel a* values. Although a* has the greatest effect on WID, the two bleaching protocols reached higher WID values than those of dentin and enamel control.

The methodology employed in this study allows us to estimate individual color changes in each hard dental tissue and dentin and enamel contributions to the overall color change. Besides the aforementioned limitations, our methodology is unable to measure the real color change caused by the staining and bleaching procedures, and causes some tissue loss during specimen sectioning. Another limitation is that applying an optical solution, such as glycerin, between tissues before measuring recombined specimens might fail in reproducing enamel-dentin junction, affecting light interaction with tooth structure.

## Conclusions

Our study found that enamel color significantly affects tooth color changes even when discoloration is caused by dentin staining. Additional application of a bleaching agent on enamel plays no role in improving internal bleaching efficacy.
